# Compensatory Neural Responses to Cognitive Fatigue in Young and Older Adults

**DOI:** 10.3389/fncir.2019.00012

**Published:** 2019-02-22

**Authors:** Immanuel Babu Henry Samuel, Chao Wang, Sarah E. Burke, Benzi Kluger, Mingzhou Ding

**Affiliations:** ^1^J. Crayton Pruitt Family Department of Biomedical Engineering, University of Florida, Gainesville, FL, United States; ^2^Department of Neuroscience, University of Florida, Gainesville, FL, United States; ^3^Department of Neurology, University of Colorado, Denver, Denver, CO, United States

**Keywords:** mental fatigue, young, old, reactive cognitive control, brain compensation, EEG, Stroop

## Abstract

Prolonged performance of a demanding cognitive task induces cognitive fatigue. We examined the behavioral and neural responses to fatigue-induced cognitive impairments in young and older adults. Particular emphasis was placed on whether the brain exhibited compensatory neural activity in response to cognitive fatigue. High-density EEG was recorded from a young (*n* = 16; 18–33 years of age) and an older (*n* = 18; 60–87 years of age) cohort who performed a Stroop task continuously for ∼2 h with no breaks. In the young cohort, behavioral performance declined as the experiment progressed, reflecting the deleterious effects of cognitive fatigue. Neurophysiologically, in addition to declining neural activity as cognitive fatigue developed, there is also evidence of region- and time-specific increase in neural activity, suggesting neural compensation. The compensatory activities followed patterns paralleling that of posterior-anterior shift in aging (PASA) and early to late shift in aging (ELSA) observed in cognitive aging and helped to moderate fatigue-induced behavioral deterioration. In the older cohort, behavioral performance did not decline as the experiment progressed, and neural activity either declined or stayed unchanged, showing no evidence of neural compensation, in contrast to the young. These results suggest that young and older adults coped with cognitive fatigue differently by exhibiting differential responses as a function of time-on-task at both the behavioral level and the neural level.

## Introduction

Compared to the appropriate control groups, cognition is generally impaired in the aged population and in patients suffering from neurological diseases. For the aged population, the appropriate control group is healthy young adults, whereas for neurological patients, the appropriate control group is gender- and age-matched healthy individuals. Neurophysiologically, task-evoked neural responses in the aging or diseased brain are generally weaker, signifying impaired brain function (Carter et al., 2001; Lorist et al., 2005; Cader et al., 2006; Cheng and Hsu, 2011). However, studies have also found brain regions where the activation is stronger in aging and in neurological diseases, and these increased activities are thought to reflect neural compensation (Cabeza et al., 2002); the neural networks associated with these compensatory activities are termed compensatory neural networks (Reuter-Lorenz and Cappell, 2008). When performing a task, these compensatory neural networks may get engaged when the primary task network is impaired, with this engagement yielding behavioral benefits (Anderer et al., 2003; Lum et al., 2009; Wang et al., 2016; Babu Henry Samuel et al., 2018).

Cognitive aging research has identified several general patterns of compensatory neural activity. For example, posterior-anterior shift in aging (PASA) reflects that impaired posterior sensory processing is associated with increased compensatory activity in higher-order anterior brain areas (Cabeza et al., 2004). Early to late shift in aging (ELSA), observed in age-related impairments during working memory tasks, reflects a reduction in brain activity during an early period of neural processing (e.g., memory retention), which is then followed by increase in brain activity in a later period of neural processing (e.g., memory retrieval) (Dew et al., 2012).

As alluded to earlier, most studies of neural compensation utilize a between-subject design, such as old-versus-young or diseased-versus-healthy. We have recently begun to examine neural compensation in a fatigue paradigm in which participants are asked to perform a demanding cognitive task for a prolonged period of time (Wang et al., 2014). This paradigm reliably induces cognitive fatigue. In young adults, with increasing cognitive fatigue, behavioral performance declined, and there is evidence of neural compensation that accompanies fatigue-induced cognitive impairments (Wang et al., 2016). To what extent the aforementioned patterns of compensatory neural activities typically associated with aging-related cognitive impairments can be generalized to neural responses to cognitive-fatigue induced impairments in the young population has not been studied. Addressing this problem is the first objective of this study.

For older adults, compensatory mechanisms are engaged at baseline, as the foregoing discussion suggests, to counteract the deleterious effects of cognitive decline in aging (Davis et al., 2008; Dew et al., 2012). Will older adults have the capacity to recruit additional resources to cope with the cognitive fatigue related impediments? Prior studies comparing young and older adults have found that, although older adults generally had slower reaction time at baseline, they did not show additional reaction time slowing during driving induced fatigue, in contrast to the young cohort who exhibited significant reaction time slowing with the development of fatigue (Philip et al., 1999). Similarly, older adults did not show any motor-related performance decline with increase in time-on-task, whereas the younger participants did (Bunce et al., 2004). These findings suggest that slower reaction time at baseline, possibly due to speed-accuracy tradeoff in aging (Smith and Brewer, 1995; Forstmann et al., 2011), is not followed by additional slowing with the onset of cognitive fatigue. How the brain responds to cognitive fatigue in the older population and whether neural compensation is behind the absence of performance deterioration has not been systematically studied. Addressing this problem is the second objective of this study.

We recruited participants in two age groups: a young cohort of *n* = 16 (18–33 years of age) and an older cohort of *n* = 18 (60–87 years of age). High-density EEG was recorded while participants performed a cued Stroop task continuously for ∼2 h without break. Neural responses evoked by target words were examined for time-on-task effects. Particular emphasis is placed on whether classical patterns of neural compensation can be observed in the young participants and whether additional neural compensation was possible in the older participants.

## Materials and Methods

### Participants

The study was approved by the Institutional Review Board of the University of Florida. A cohort of 16 healthy young adults (18–33 years of age, 9 females) and a cohort of 18 healthy older adults (60–87 years of age, 7 females) provided written informed consent and participated in the study. Participants were right-handed, native English speakers, free from neurological disorders and with normal to corrected-to-normal vision. Participants were asked to refrain from consuming caffeine or nicotine on the day of testing. All experiments began at 9.00 AM. The experimental procedure and data analysis methods, to be detailed below, were exactly the same for the two cohorts.

### Procedure

After a brief practice session to familiarize with a computerized cued Stroop task, the participants were fit with EEG electrodes and asked to perform the task continuously for 180 min (3 h). The recording was done in an electrically and acoustically shielded room to reduce contaminations by external sounds, electrical line noises and other electromagnetic interferences. Breaks for any purpose were taken only upon request and resulted in discontinuation of the experiment. In the young cohort, nine participants performed the entire 180 min of the task; 5 completed at least 160 min. One participant quit before 160 min; this subject was rejected. Another participant exhibited excessive body motion and was also rejected. The data from the 14 young subjects were analyzed here and the time-on-task varied from 0 to 160 min. In the older cohort, 11 participants performed the entire 180 min of the task; 6 completed at least 100 min. One participant quit before 100 min and was rejected. Another participant was also rejected due to poor EEG data quality. The data from the remaining 16 older subjects were analyzed here and the time-on-task varied from 0 to 100 min.

### Experimental Paradigm

**FIGURE 1:**
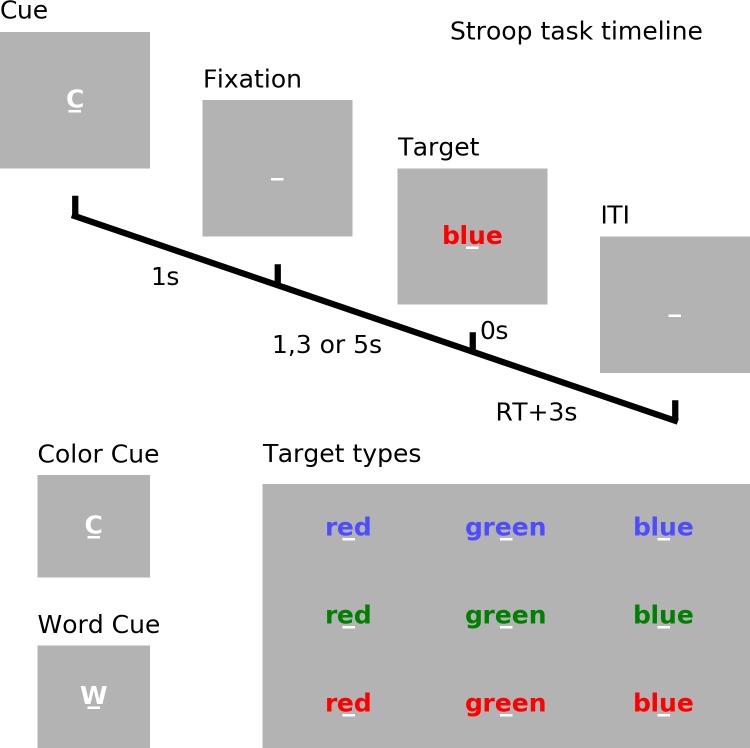
Experimental paradigm. Timeline of the cued Stroop task. Depicted is a color incongruent trial. Time 0 denotes the onset of the target word. The correct response is red. ITI, inter-trial interval. RT, reaction time.

See Figure 1. Each trial began with a cue (‘w’ for word or ‘c’ for color) which lasted for 1 s. After a random cue-target interval of 1, 3, or 5 s, one of three target words (‘red’, ‘blue,’ or ‘green’) printed in different font colors (red, blue, or green) appeared on the screen. If the trial was cued for word (word trial), participants were instructed to read the word; if the trial was cued for color (color trial), participants were instructed to name the color of the font in which the word was printed. When the font color of the word and the meaning of the word matched, it is a congruent trial; otherwise, it is an incongruent trial. Reaction time (RT) was determined by a voice activated microphone and the pronounced word was manually recorded by the experimenter. The next trial started 3 s after the voice response.

### EEG Recording and Preprocessing

The EEG data was recorded using a 128-channel BioSemi Active Two System. Positions of the electrodes were obtained with a Polhemus spatial digitizer. Offline preprocessing was performed using BESA 5.3, EEGLAB and custom Matlab scripts. Data segments contaminated by movements were rejected. The remaining data was bandpass filtered between 0.1 and 83 Hz, down-sampled to 250 Hz, and epoched from -1.5 to 1 s relative to target word onset (0 s). After rejecting noisy trials, independent component analysis (ICA) was applied to remove artifacts resulting from eye movements, eye blinks, muscle activity, and line noise. After ICA, any epoch with incorrect behavioral response or with voltage exceeding 75 μV in any scalp channel was further rejected from analysis. Artifacts-corrected data was then re-referenced against the average reference. Inter-individual differences in electrode positioning were controlled for by using a spherical spline interpolation to project 128 channel preprocessed data onto the standard 81 channel montage (10–10 montage) implemented in BESA.

### EEG Analysis and Interpretation

Similar to our previous paper on cue-related neural activity based on the young cohort data (Wang et al., 2016), we mainly focused on the more cognitively demanding incongruent trials where the word and its font color conflicted, thereby requiring more cognitive control to resolve the conflict and to suppress the irrelevant response. An example of such a trial was illustrated in Figure 1. Because of the high cognitive demand, these trials were more prone to the deleterious effects of cognitive fatigue, and were thus suited for analyzing the effects of cognitive fatigue on neural responses. The time period between -200 and 0 ms relative to target word onset was demeaned when calculating the event-related potentials (ERP).

Based on prior ERP studies of the Stroop task, we selected two times of interest (TOI) for analysis: (1) the Early Period (150–300 ms), in which sensory and early attentional processes are active (Weinstein, 1995; Atkinson et al., 2003); and (2) the Late Period (300–1000 ms), in which higher-order attention and conflict-related processing are active (West, 2003; Larson et al., 2004; Perlstein et al., 2006), along with response-related cognitive adjustments and adaptation processes (Grune et al., 1993; Liotti et al., 2000).

As the experiment progressed (i.e., as time-on-task increased), the ERP responses in the early period and late period of stimulus processing were expected to undergo dynamic change due to increasing cognitive fatigue. To determine the time-on-task effect on ERP during each TOI, a moving-window approach across trials was adopted, in which the ERPs were estimated within a block of 40-min in duration and the block was stepped forward with a 10-min increment; there was a total of 13 blocks for the young cohort and a total of 7 blocks for the older cohort. These parameters were chosen for the following reasons. (1) 40-min time blocks allowed us to get a stable estimate of ERP with well-defined components. In fatigue paradigms, despite instructions telling the subjects not to move, they do move because cognitive fatigue makes them uncomfortable. Sufficient number of trials was needed to overcome the negative effects of movement and other noises. Compared to 40 min blocks, ERPs obtained from 10 min blocks were not stable, and the estimates of the ERP component amplitudes were too susceptible to noise contamination. (2) Using 40 min blocks without overlapping would reduce the temporal resolution needed to examine the detailed evolution of ERP responses with fatigue. Therefore, a 30 min overlap was used to achieve the best compromise between stable ERP estimation and good temporal resolution. For each EEG channel, a mixed-effects model was applied to examine whether ERP amplitudes during each TOI demonstrated significant time-on-task effects (thresholded at *p* < 0.05, controlling for multiple comparisons with false discovery rate). Channels were then grouped into regions of interest (ROIs) based on the time-on-task modulation patterns of the ERPs.

Event-related potentials’ time-on-task effects were expected to fall broadly into three categories: (i) amplitude decreasing with time-on-task, (ii) amplitude increasing with time-on-task, and (iii) amplitude staying unchanged (Boksem et al., 2005). The first category was taken to signify impairment and the second category compensation. The relation between ERPs in different TOIs and ROIs and increasing time-on-task was subjected to a linear regression analysis and the slopes from such analysis gave the rates of ERP changes. The relationship between the compensation related increase and the impairment related decrease of ERPs in different TOIs and ROIs were examined using these rates of changes.

### Relationship Between Compensatory Neural Response and Behavior

We quantified the time-on-task changes of RT, error rate and coefficient of variation of reaction time (CVRT) using the same moving block approach described above to be consistent with the ERP analysis. The change in these behavioral measures was tested using paired *t*-test between the values obtained in the first 40-min time-block and the values obtained in each consecutive 40-min time-block (corrected for multiple comparisons using False Discovery Rate – FDR). Although mean RT within each block is a well-established measure to quantify behavioral performance, it does not capture other aspects of the performance, such as RT variability. RT variability is reflective of attention fluctuations (Allcock et al., 2009), especially ‘attention lapses’ (Tamm et al., 2012), which are indicative of a fatigued mental state (Bruce et al., 2010). Our past work has shown that the CVRT, defined as the ratio of the standard deviation of reaction time to the mean of reaction time, was a suitable measure for characterizing fatigue-related behavioral change (Wang et al., 2014). Association of compensatory ERP change with change in behavioral performance was analyzed using a multiple regression model in which the independent variables were the rates of ERP changes in different TOIs and ROIs and the dependent variable was the rate of change of CVRT. Effect sizes, where appropriate, were reported to supplement statistical significance testing (Rosenthal and Rosnow, 1991).

## Results

### Cognitive Fatigue and Behavioral/Neural Responses in Young Adults

#### Behavior

**FIGURE 2:**
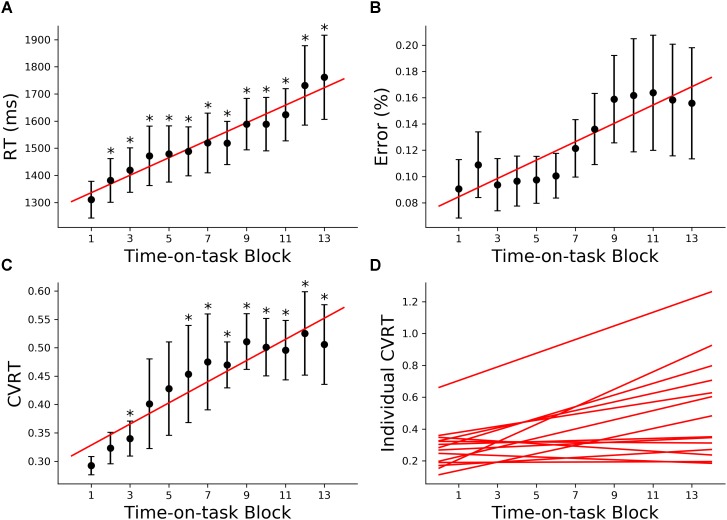
Behavioral analysis (young adults). **(A)** Group average reaction time (RT), **(B)** group average error rate, **(C)** group average coefficient of variation of reaction time (CVRT), **(D)** individual regression fits to CVRT as a function of time-on-task blocks. The slopes of the regression lines denoted the rate of behavioral change with cognitive fatigue. ^∗^*p* < 0.05, corrected for multiple comparisons using False Discovery Rate (FDR).

The 160 min of task performance was divided into 40-min time blocks stepped forward with 10 min increments. The RT and CVRT obtained for successive time blocks showed significant increase compared to the first time block (Figure 2A,C). Error rate showed an increasing trend but the increase did not reach statistical significance (Figure 2B). In the subsequent analysis we mainly focused on the change in CVRT because CVRT has been shown to be a good behavioral measure for characterizing cognitive fatigue (Wang et al., 2014). Linear regression model was fit to the CVRT across time-on-task blocks for each subject. The positive slopes in Figure 2D demonstrated the progressive deterioration of the task performance.

#### Neural Activity: Regions of Interest

**FIGURE 3:**
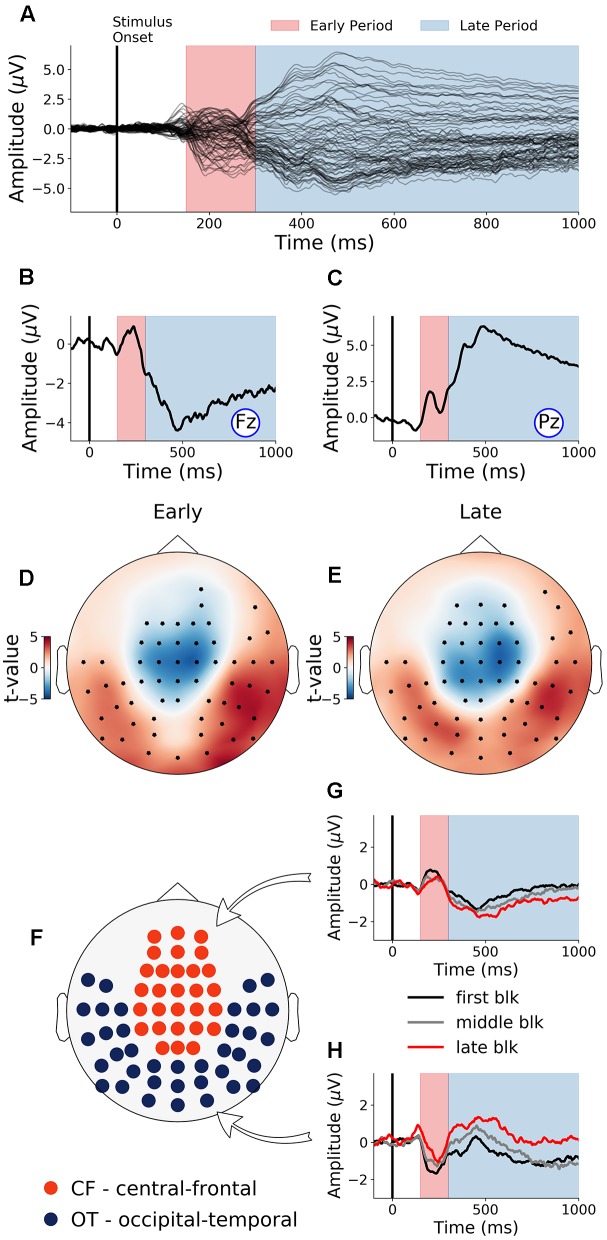
Event-related potentials (ERP) and effects of time-on-task (young adults). **(A)** ERPs during baseline time block (first 40 min) from all channels. **(B,C)** Baseline ERPs from Fz and Pz. **(D,E)** Topographies showing channels where ERP amplitudes were significantly modulated by time-on-task (*p* < 0.05, FDR corrected) for the early period and the late period of stimulus processing. **(F)** ROIs defined based on analysis in **(D,E)**. **(G,H)** ERPs from first, middle, and last time-on-task blocks from central-frontal ROI and occipital-temporal ROI.

The first 40 min, which was the first time block of the experiment, was defined as the baseline. During this baseline time block, the grand average ERPs evoked by the target word were shown for all electrodes in Figure 3A, where the two TOIs were marked using colored shading. ERPs from Fz and Pz were shown in Figure 3B,C. Stepping forward across time blocks, the time-on-task effect analysis on ERP amplitude, shown as scalp topographies in Figure 3D,E, revealed that there were two groups of electrodes for which the ERP amplitudes were systematically modulated by time-on-task for each of the two TOIs. These two groups of electrodes, located in the occipital-temporal sites and the central-frontal sites, formed the two ROIs (Figure 3F). ERPs from these two ROIs during the first block (baseline), the middle block (block 7), and the last block (block 13) are shown in Figure 3G,H to illustrate the time-on-task effect on ERP amplitude during the early and late period of stimulus (target word) processing. For early stimulus processing (early period), as time-on-task progressed, the ERP amplitudes decreased in both ROIs. For late stimulus processing (late period), the ERP amplitudes decreased in occipital-temporal ROI, but increased in central-frontal ROI (i.e., a negative ERP component became more negative).

#### Neural Activity: Effects of Cognitive Fatigue

##### Early period of stimulus processing

**FIGURE 4:**
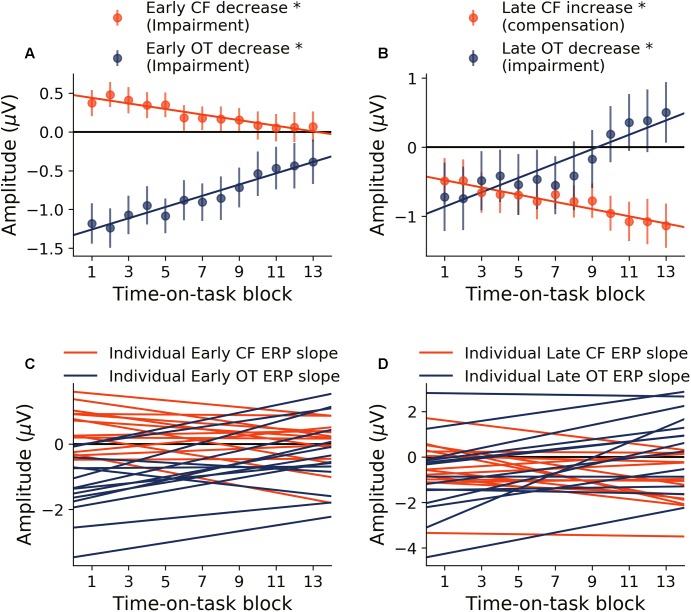
Time-on-task effect on ERP (young adults). **(A)** ERP amplitude indexing the early processing of target words decreased in both central-frontal (CF) ROI and occipital-temporal (OT) ROI. **(B)** ERP amplitude indexing late target processing decreased for occipital-temporal ROI but increased in central-frontal ROI. **(C,D)** Linear regression lines were fit to ERP changes over time-on-task blocks for each participant. Slopes of these regression lines were taken as the rates of ERP changes with time-on-task. ^∗^*p* < 0.05, FDR corrected.

Neural activity during the early period of target word processing mainly reflects sensory processing and early attention-related activity (Hoffman, 1990; Luck, 1995; Nieuwenhuis et al., 2003). As shown in Figure 4A, with increase in time-on-task, ERP amplitude in the early period underwent steady decrease in both occipital-temporal and central-frontal ROI (*p* < 0.05, FDR corrected), suggesting progressive impairments of sensory, early attentional and target discrimination processes. Linear regression fit to the ERP amplitude changes were shown in Figure 4C to demonstrate individual variability.

##### Late period of stimulus processing

Neural activity during the late period of target word processing reflected higher-order processes including conflict detection/resolution and cognitive adjustments (Grune et al., 1993; Liotti et al., 2000). ERPs during the late period exhibited a more complex pattern of temporal dynamics in response to cognitive fatigue. In the occipital-temporal ROI, ERP amplitude decreased with time-on-task increase (*p* < 0.05, FDR corrected), whereas the central-frontal ERP amplitude increased with time-on-task increase (*p* < 0.05, FDR corrected), as shown in Figure 4B. This result suggests that while some regions underwent progressive impairment due to the onset and deepening of cognitive fatigue, reflected by decrease in ERP amplitude, other areas showed progressive recruitment of compensatory activities, reflected by the increase in ERP amplitudes. The individual slopes of the linear regression fit to ERP amplitude change were shown in Figure 4D to demonstrate individual variations.

#### Relation Between ERP Rates of Change

**FIGURE 5:**
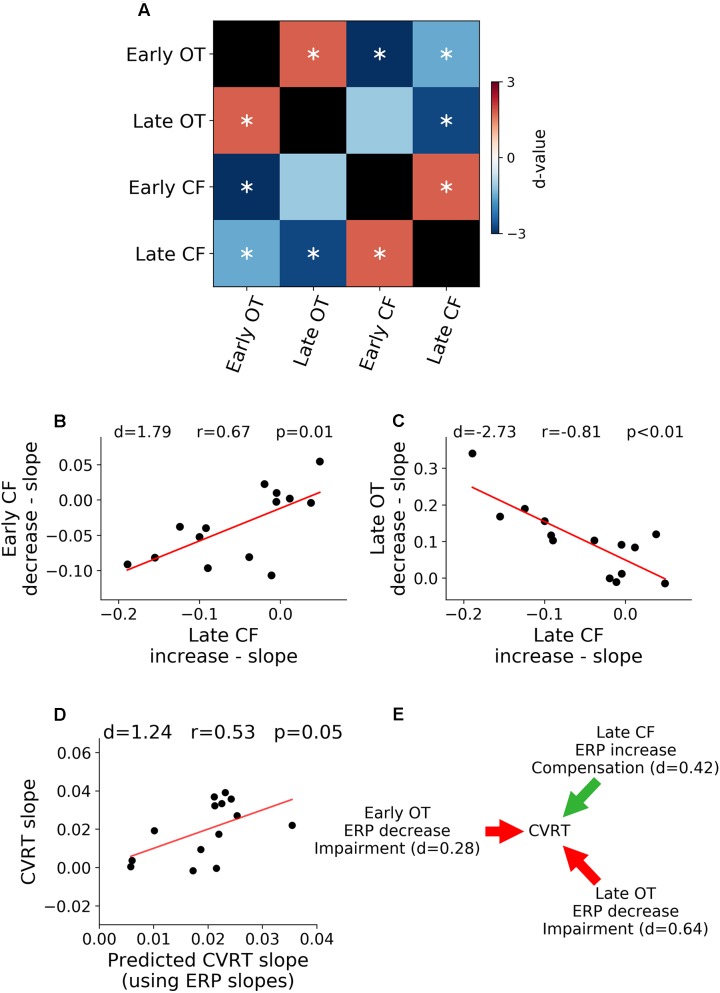
Relation between neural activity changes and performance. **(A)** Pairwise correlation between ERP regression slopes across ROIs and TOIs. **(B)** Late ERP increase was correlated with early ERP decrease in central-frontal (CF) ROI (ELSA). **(C)** Late occipital-temporal (OT) ERP decrease was correlated with late central-frontal (CF) ERP increase (PASA). **(D)** Rates of ERP changes over time-on-task across different TOIs and ROIs predict the rate of behavioral change over time-on-task. **(E)** Illustration of functional contributions of each ERP rate of change to the rate of behavioral change. ^∗^*p* < 0.05, FDR corrected.

Relations between rates of ERP amplitude changes in different TOIs and ROIs were shown in Figure 5A. The rates of neural activity changes were generally significantly correlated (*p* < 0.05, FDR corrected). In particular, in Figure 5B, the decrease in early ERP amplitude and the increase in late ERP amplitude in the central-frontal ROI were shown to be significantly correlated (*r* = 0.67, *d* = 1.79, *p* < 0.05, FDR corrected, Figure 5B), demonstrating an ELSA-like compensation pattern observed in prior cognitive aging research (Davis et al., 2008; Dew et al., 2012). In Figure 5C, the rate of occipital-temporal ERP decrease (posterior decrease) and central-frontal ERP increase (anterior increase) during the late period was significantly correlated (*r* = -0.81, *d* = -2.73, *p* < 0.05, FDR corrected). This posterior decline followed by anterior increase is similar to the PASA compensation pattern observed in prior cognitive aging research (Davis et al., 2008).

#### Relation Between Neural Activity and Behavior

A multiple regression analysis was applied to examine the relation between neural changes and behavioral changes. The rate of change of ERP over time-on-task across the two ROIs and two TOIs were treated as independent variables and the rate of change of CVRT as the dependent variable. As shown in Figure 5D, the rates of ERP changes predicted the rate of behavior change (*d* = 1.24, *r* = 0.53, *p* = 0.05), demonstrating functional significance of neural changes over the course of the experiment. To further characterize the effect of each ERP rate of change, the model coefficients were analyzed, and the results shown in Figure 5E. For both early (*d* = 0.28) and late ERPs (*d* = 0.64) in occipital-temporal ROI, the higher the rate of ERP amplitude decrease with time-on-task (signaling faster fatigue-related neural decline), the higher the rate of increase of CVRT (signaling faster fatigue-related behavioral decline). In contrast, for the late ERP in the central-frontal ROI (*d* = 0.42), the higher the rate of ERP amplitude change (signaling faster fatigue-related recruitment of compensatory neural resources), the slower the rate of increase of CVRT (signaling slower fatigue-related behavioral decline). The sizes of these effects (Cohen’s *d*) were small to moderate. The change of the early central-frontal ERP did not have an effect on the change of CVRT (*d* < 0.2).

### Cognitive Fatigue and Behavioral/Neural Responses in Older Adults

#### Behavior

**FIGURE 6:**
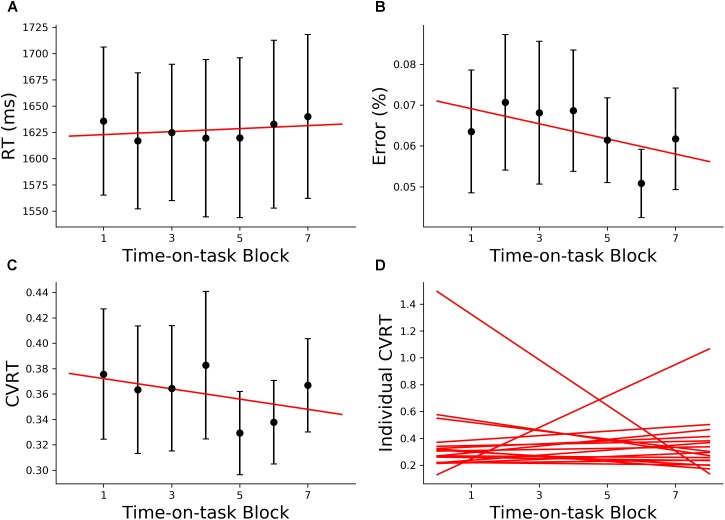
Behavioral analysis (older adults). **(A)** Group average reaction time (RT), **(B)** group average error rate, **(C)** group average CVRT, **(D)** individual regression fits to CVRT as a function of time-on-task blocks. The slopes of the regression lines denoted the rate of behavioral change with cognitive fatigue. ^∗^*p* < 0.05, corrected for multiple comparisons using False Discovery Rate (FDR).

Unlike the young cohort, the older cohort showed no significant change in RT, error rate or CVRT with increase in time-on-task, as shown in Figure 6A–C. This finding is in agreement with prior fatigue research showing that older adults were able to maintain a consistent level of performance over an extended period of time (Falkenstein et al., 2002). At the individual subject level, there was significant variability in the rate of behavioral change (Figure 6D), as expected.

#### Neural Activity: Effects of Cognitive Fatigue

**FIGURE 7:**
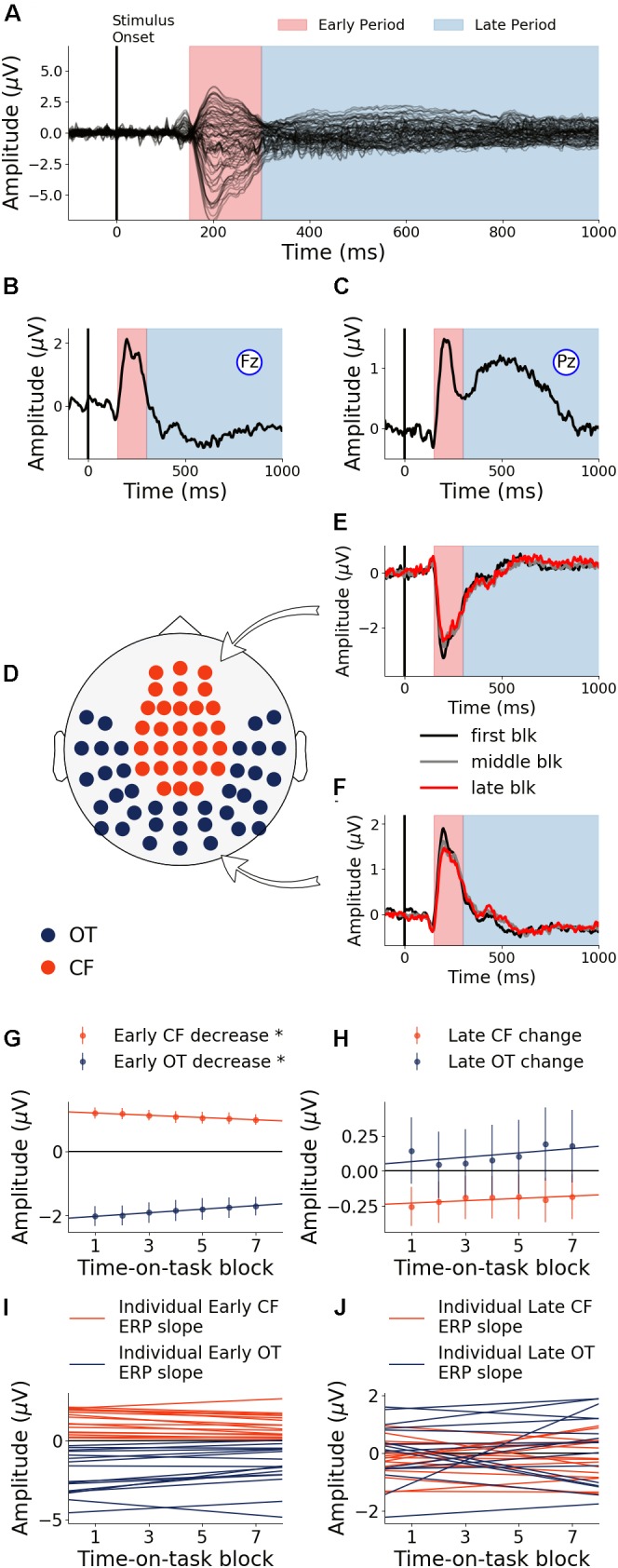
Event-related potentials and effects of time-on-task (older adults). **(A)** ERPs during baseline (first 40 min) from all channels. **(B,C)** Baseline ERPs from Fz and Pz. **(D)** ROI definition. **(E,F)** ERPs from first, middle and last time-on-task blocks from central-frontal (CF) ROI and occipital-temporal (OT) ROI. **(G)** ERP amplitude during early target processing decreased in both central-frontal ROI and occipital-temporal ROI. **(H)** ERP amplitude in late target processing did not show significant change with time-on-task. **(I,J)** Linear regression lines were fit to ERP changes over time-on-task blocks for each participant. Slopes of these regression lines were taken as the rates of ERP changes with time-on-task. ^∗^*p* < 0.05, FDR corrected.

Baseline ERPs (first 40 min) from all channels and from Pz and Fz were shown in Figure 7A–C. The early and late TOIs associated with stimulus processing were defined in the same way as the young for consistency and comparability. The two ROIs were also similarly defined (Figure 7D). ERPs from the two ROIs for three different time-on-task blocks were shown in Figure 7E,F. Statistical analysis revealed that ERP amplitudes from the early TOI declined significantly with increase in time-on-task in both ROIs (*p* < 0.05, FDR corrected, Figure 7G). ERP amplitudes from the late TOI showed no significant change as a function of time-on-task (*p* = 0.36, Figure 7H). Like the young cohort, early target-evoked response decreased with the development and deepening of cognitive fatigue, suggesting progressively impaired sensory and early attention-related processing. Unlike the young cohort, no increase in late target-evoked response was observed, suggesting a lack of compensatory neural activity in the older cohort. Individual variability in changes in neural responses can be seen in Figure 7I,J.

## Discussion

Prolonged performance of a cognitively demanding task can lead to cognitive fatigue. The adverse behavioral consequences of cognitive fatigue have been well studied in the literature (Akerstedt et al., 2004; Boksem et al., 2005; Boksem and Tops, 2008; Cheng and Hsu, 2011; Hopstaken et al., 2016). Here, we examined the behavioral and brain responses to cognitive fatigue in both young and older adults. Analyzing EEG data from the subjects performing a Stroop task continuously for at least 2 h and 40 min for young subjects and 1 h and 40 min for older subjects we reported the following findings. In the young cohort, performance declined with increase in time-on-task. Early ERP components in both the occipital-temporal and central frontal ROIs declined as well with increase in time-on-task, indicating fatigue-related impairments in sensory and early attentional processing. Whereas late ERP components in occipital-temporal ROI decreased with increase in time-on-task, late ERP components in the central-frontal ROI increased with increase in time-on-task, suggesting posterior impairments of neural processing that were accompanied by frontal neural compensation. The rates of amplitude changes of different ERP components over different TOIs and ROIs were related with one another, with the patterns being reminiscent of PASA and ELSA reported in cognitive aging (Cabeza et al., 2002, 2004). Importantly, these rates of ERP changes predicted the rate of change in behavioral performance (CVRT) over the whole experiment in a linear model, and an analysis of the model coefficients suggested that the declining ERPs (impairment) were detrimental for the maintenance of task performance whereas the increasing ERPs (compensation) were beneficial for the maintenance of task performance. In the older cohort, there was no significant change in behavioral performance across the entire experiment. While the early ERP components declined in both ROIs, the late ERP components exhibited no systematic change as a function of time-on-task; thus only neural impairment, but no compensatory neural increase, was observed in the older adults.

### Patterns of Neural Decline and Neural Compensation

To separately examine the impact of cognitive fatigue on sensory and early attentional processes (Hoffman, 1990; Atkinson et al., 2003) and on higher order activities such as cognitive control and conflict processing (Botvinick et al., 2001), we separated target word evoked activity into two time periods, namely: (1) Early Period (150–300 ms) and (2) Late Period (300–1000 ms). In the healthy young cohort, during the early period, ERP topographies highlight the involvement of the occipital-temporal region (Patel and Azzam, 2005) and the central-frontal region (Huster et al., 2010). With the onset and deepening of cognitive fatigue, ERPs in the early period underwent a steady decrease, suggesting that sensory and early attentional processes were becoming increasingly impaired (Figure 3D). This is reminiscent of prior research showing that early visual processing is very susceptible to the effects of aging (Davis et al., 2008; Dew et al., 2012) and neuropathology (Uc et al., 2005; Armstrong, 2011; Weil et al., 2016). However, in these prior studies, aging and neuropathology related visual decline has other contributing factors such as corneal damage, decrease in vascular density and the reduced number of rods (Schneider and Pichora-Fuller, 2000). In subjects undergoing progressive cognitive fatigue over a short time scale (a few hours compared to a few decades), however, such anatomical changes are unlikely; therefore, the progressive decline of neural activities during the early period reflects neural impairments, free from the confounding influences of factors such as corneal/retinal changes. Late period ERPs are associated with higher-order cognitive processes such as conflict processing and response adjustments (Liotti et al., 2000; Atkinson et al., 2003; Larson et al., 2009). With the onset and deepening of cognitive fatigue, ERPs over the occipital-temporal region during the late period steadily decreased (Figure 3E), demonstrating the deleterious effects of cognitive fatigue on the underlying neural activities. In contrast to ERP decrease in both ROIs during the early period and in the occipital-temporal ROI during the late period, ERP amplitude increased with increase in time-on-task in the central-frontal region during the late period, indicating neural compensatory activity.

Early to late shift in aging (ELSA) is a commonly observed pattern in cognitive compensation in aging and in neurological disorders (Dew et al., 2012). In ELSA, decline in early neural processing is compensated by increase in late neural processing. The data from the young cohort undergoing cognitive fatigue exhibited the ELSA pattern, namely, early period neural activity decline is accompanied by the late period neural activity increase. To test whether early neural processing impairments and late neural compensation is related, we compared the rate of increase and rate of decrease in ERP amplitudes across the two time periods, and found that within the central-frontal ROI, ERP decrease in early period were correlated with the ERP increase in late period, suggesting that the magnitude of compensation is proportional to the magnitude of impairments. Similar effects have been observed in a previous report on cognitive fatigue (Hopstaken et al., 2016), although in that report, the neural compensatory activity was not explicitly recognized.

Another form of compensatory response on cognitive aging is posterior-anterior shift in aging (PASA). In PASA, declined posterior processing is accompanied by increased anterior compensatory activity (Grady et al., 1994; Cabeza et al., 2004; Davis et al., 2008). The decrease in posterior/occipital activity is thought to reflect sensory decline as a function of age (Lindenberger and Baltes, 1994). Our results demonstrated that PASA can be observed in a cognitive fatigue paradigm as well, namely, the occipital-temporal ERP decrease was accompanied by a central-frontal ERP increase, and the rates of ERP decrease and increase in different regions of the scalp are correlated with one another, suggesting that sensory decline can occur acutely as a result of cognitive fatigue, and the frontal compensatory activity can be recruited acutely with the rate of recruitment being proportional to the rate of impairment of posterior neural activity (Davis et al., 2008).

In the older cohort, we only observe ERP decline in the early target evoked response period but no ERP increase in any time period or regions of interest, suggesting that cognitive fatigue caused further deterioration of sensory and early attentional processes but did not trigger neural compensatory response. It is worth noting that the decline in ERP amplitude in the older cohort is not as drastic as that seen in the young cohort, which along with the lack of performance decline, appears to suggest that the old subjects are better able to resist the impairing effects of cognitive fatigue (Falkenstein et al., 2002). Whether the lack of compensatory activity is due to capacity limitation or other factors such as the lack of necessity to compensate is difficult to ascertain in the current paradigm.

### Relating Neural Changes and Behavioral Changes

Cognitive fatigue results in performance decline in young adults. This has been consistently reported in the literature (Hopstaken et al., 2016; Wang et al., 2016). In this paper, we further reported that concomitant with performance decline, neural changes also took place. To what extent neural changes and behavioral changes are related determines the functional significance of the observed neural activity changes. Past research has not always found a relationship between the two. Smith et al. (1999) showed that although the brain activity was significantly reduced for individuals with high risk for Alzheimer’s disease compared to healthy controls, there were no performance differences in both visual naming and letter fluency tasks (Drummond et al., 2000). This may partly explain why we did not see performance decline in the older cohort despite impairment in neural responses. Moreover, increased neural activity in aging and in brain disorders, which were interpreted as compensatory activity, has been found to be associated with reduction in performance (Logan et al., 2002; Cabeza and Kingstone, 2006; Wingfield and Grossman, 2006), a finding at variance with the expectation that neural compensation should benefit behavioral performance. With these questions in mind, we performed a multiple regression analysis with neural changes as independent variables and behavioral change as dependent variables for the young cohort, given this cohort’s rich impairment-versus-compensation dynamics. In addition to showing that neural changes predicted behavioral changes, our results further demonstrated that decreasing ERP amplitudes (impairments) are associated with worsening of task performance, whereas increasing ERP amplitude (compensation) helps to moderate performance decline.

### Limitations

This study has a number of limitations. The first is the small sample size for both cohorts. Future studies will recruit larger numbers of participants to test the robustness of the findings reported here. The second is the different length of time-on-task used in the analysis for the two cohorts (at least 2:40 h for young adults and at least 1:40 h for older subjects). Although the task was designed to be performed for a maximum of 3 h for both cohorts, the participants in the old cohort had a higher tendency to quit earlier. The third is that the paradigm, designed to be more applicable to real world scenarios where people pace their responses based on their cognitive state, allowed self-pacing. To what extent self-pacing increased with the increase in time-on-task cannot be ascertained. The fourth is that the educational level of the participants was not acquired. Because education is known to contribute to cognitive reserve which in turn affects the brain response to cognitive fatigue (Jongen et al., 2012), without information on education, the relationship between cognitive reserve, cognitive fatigue and compensation cannot be addressed in this study. The fifth is that although both the young and older cohorts developed the sensation of cognitive fatigue as the experiment progressed, per post-experiment conversations, we did not have information to quantify the change of this sensation as a function of time-on-task (Burke et al., 2018).

## Summary

In this study we analyzed the behavioral and neural responses to cognitive fatigue induced impairment in young and older adults. In young adults, we found that prolonged performance of a cognitively demanding task led to declined behavioral performance and was associated with both impaired neural responses as well as neural compensation. The spatial and temporal patterns of neural impairments and neural compensation were consistent with two widely reported patterns of neural compensation in the cognitive aging literature, namely, ELSA and PASA. In the older adults, behavioral performance did not decline, and there were only impaired neural responses but no compensatory neural activity.

Compensatory neural processes have been traditionally studied in cross-sectional designs where populations with advanced age or brain disorders are compared with young or gender- and age-matched healthy populations. In such comparisons it is difficult to determine whether neural compensation is developed over an extended period of time or can be recruited acutely. Our study suggests that the cognitive fatigue paradigm in healthy adults (both young and older) may be useful as a model to induce neural impairments and compensatory processes in a short period of time and provide an opportunity to study the functional roles of these processes in maintaining task performance.

## Ethics Statement

This study was carried out in accordance with the recommendations of the NIH Protection of Human Research Subjects and HIPPA for research guidelines with written informed consent from all subjects. All subjects gave written informed consent in accordance with the Declaration of Helsinki. The protocol was approved by the University of Florida Gainesville Institutional Review Board.

## Author Contributions

MD and BK contributed conception and design of the study. CW and IBHS organized the database, performed the statistical analysis, and wrote the first draft of the manuscript. MD, BK, CW, and SB wrote sections of the manuscript. All authors contributed to manuscript revision, read and approved the submitted version.

## Conflict of Interest Statement

The authors declare that the research was conducted in the absence of any commercial or financial relationships that could be construed as a potential conflict of interest.
